# Single Intravitreal Aflibercept Injection for Unilateral Acute Nonarteritic Ischemic Optic Neuropathy

**DOI:** 10.1155/2015/783241

**Published:** 2015-01-08

**Authors:** Ziya Ayhan, Gamze Kocaoğlu, Aylin Yaman, Meltem Söylev Bajin, A. Osman Saatci

**Affiliations:** Department of Ophthalmology, Dokuz Eylül University, 35320 İzmir, Turkey

## Abstract

Acute nonarteritic ischemic optic neuropathy (ANAION) is the most common optic neuropathy in the elderly population without a well-established treatment. A 67-year-old man with a sudden painless visual loss in his left eye of one-day duration was diagnosed to have left ANAION. Next day, 2 mg aflibercept injection was injected intravitreally in OS. Visual acuity improved to 7/10 from 1/10 a week after the injection. Mean retinal nerve fiber layer thickness (RNFLT) was reduced to 159,7 *μ*m from 182,4 *μ*m at the first week. Visual fields improved dramatically during the follow-up of three months. The aim of this study is to present a case having ANAION treated with a single intravitreal aflibercept injection and discuss the place of intravitreal anti-VEGF injections in the treatment of armamentarium of ANAION.

## 1. Introduction

Acute nonarteritic anterior ischemic optic neuropathy (ANAION) is the most common type of optic neuropathy characterised with a sudden visual deterioration in the elderly population [1]. There have been no prospective randomised-controlled studies to show a therapeutic benefit for any treatment modality [2–4]. However, in 2008, Hayreh and Zimmerman [5] demonstrated that early treatment of ANAION with 80 mg oral prednisolone therapy improved both the visual acuity and visual field in their nonrandomised-controlled study. Some physicians injected various doses of triamcinolone acetonide intravitreally in eyes with ANAION instead of systemic steroids in order to avoid side effects of the systemic steroid with some anecdotal success [6–8]. On the other hand, intravitreal antivascular endothelial growth factor (VEGF) agents may be theoretically helpful as they reduce vascular permeability and thereby decrease optic nerve head oedema [9–12].

In this case report, we report the satisfactory visual outcome obtained after the intravitreal injection of 2 mg aflibercept in a patient with unilateral ANAION.

## 2. Report of a Case

**Figure 1:**
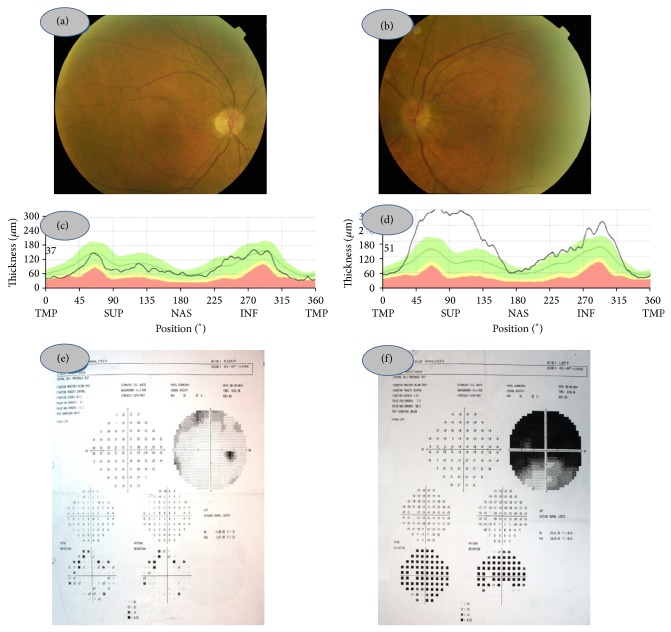
Colour fundus picture of the right eye (a) and left eye (b), retinal nerve fiber layer thickness in OD (c) and OS (d), and visual fields in OD (e) and OS (f) at the initial examination prior to left intravitreal aflibercept injection.

A 67-year-old man was referred to us with a sudden painless visual loss in his left eye of one-day duration. He had a long history of Hashimoto thyroiditis and congenital colour blindness. On our examination, left afferent pupillary defect was present. Colour vision was 8/21 in OD and 0/21 in OS with Ishihara pseudoisochromatic plates. Slit-lamp examination was unremarkable OU. Intraocular pressure was within normal limit bilaterally. Left optic disc was hyperemic and partly swollen while right optic disc had no cup (Figures 1(a) and 1(b)). Mean RNFLT was measured with Heidelberg Spectralis OCT as 89,3 *μ*m for OD (Figure 1(c)) and 182,4 *μ*m for OS (Figure 1(d)). No macular change was noted in the OCT sections OU. Right visual field was full (Figure 1(e)) and there was an upper altitudinal scotoma in the left eye (Figure 1(f)).

**Figure 2:**
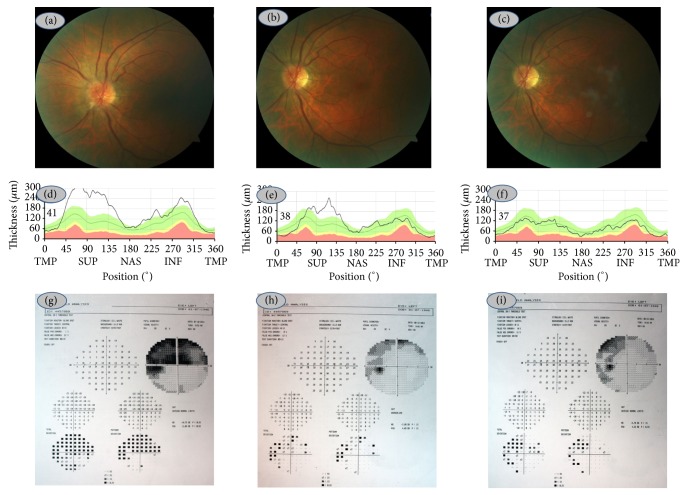
Left eye, colour fundus picture at the first week (a), first month (b), and third month (c), retinal nerve fiber layer thickness at the first week (d), first month (e), and third month (f), and visual fields at the first week (g), first month (h), and third month (i).

C-reactive protein was 2,9 mg/L and erythrocyte sedimentation rate was 10 mm/h. Our diagnosis was left ANAION. Natural course of the disease and treatment options were discussed with the patient and intravitreal anti-VEGF injection was deemed to be the choice of treatment. 2 mg aflibercept was given to the left eye under topical anesthesia next day. Best-corrected visual acuity was 7/10 a week later, 10/10 at the first month, and 10/10 at the third month. He could read 2/21 of plates a week later, 4/21 of plates at the first month, and 4/21 at the third month with Ishihara test. Visual fields improved markedly. Color fundus picture, RNFLT analyses, and visual field tests could be seen in Figures 2(a), 2(b), 2(c), 2(d), 2(e), 2(f), 2(g), 2(h), and 2(i). No injection-related complication was noted during the follow-up of three months.

## 3. Discussion

Spontaneous visual acuity improvement has been observed in 43% of patients with ANAION within six months of disease onset, but there was no visual improvement in 45% of patients and further visual deterioration was noted in 12% of cases in the control group of ischaemic optic neuropathy decompression trial [4]. Therefore, it is meaningful to search for effective therapeutic agents to improve the visual outcome. Anti-VEGF agents are being widely used in many disease processes. Anti-VEGF agents may have two positive effects on the course of the disease and thereby visual acuity. One is the lessening of the optic disc oedema by decreasing vascular permeability and the second is reducing the concomitant subretinal fluid when present [13]. In a recent clinical study, subretinal fluid was noted in approximately 10% of ANAION cases [14] and, in a recent experimental study, subretinal fluid was found to be common in a murine photochemical thrombosis model of NAION [15].

Rootman et al. [16] looked for the efficacy of 1.25 mg intravitreal bevacizumab injection on the natural history of ANAION in their nonrandomised-controlled clinical trial. Twenty-five patients were enrolled (17 eyes were treated and 8 eyes served as control) in their study. They found no difference between the injection and control group in regard to the change in visual field, visual acuity, or RNFL thickness measured with Cirrus OCT. Furthermore, two of their 17 treated eyes experienced a second ANAION episode during the follow-up.

Our preliminary report about four eyes treated with 0.5 mg intravitreal ranibizumab showed promising results after a follow-up of three months. All of our patients experienced some degree of visual gain [11]. We conducted another retrospective clinical analysis on 17 eyes of 16 patients who experienced a visual loss with a duration of 15 days or less who received 0.5 mg intravitreal ranibizumab injection. Some visual gain was noted in 14 of 17 studied eyes. In two eyes visual acuity was minimally reduced and no change was noted in the remaining eye. While preinjection mean best-corrected visual acuity (BCVA) was 1.45 ± 0.88 logMAR unit, postinjection mean BCVA was 1.00 ± 0.68, 0.86 ± 0.70, 0.80 ± 0.71, and 0.77 ± 0.70 logMAR unit, respectively, at the first week, first month, third month, sixth month, and first year. In all patients, the mean RNFLT dramatically decreased after the injection during the course of follow-up [17].

However, there are some controversial issues on the place of intravitreal anti-VEGF agent in eyes with ANAION. First is the timing of the injection after the acute episode. Animal studies suggested that therapeutic window for ANAION may be as long as 2-3 weeks [14]. Second is the possible role of anti-VEGF agents by themselves on inducing or facilitating acute NAION [18–21]. Mansour et al. [21] suggested that potential mechanisms include the vasoconstrictor effect of the anti-VEGF agents, an increase in intraocular pressure from the intravitreal injection, and the exacerbation of systemic hypertension from the stress of the procedure. Thirdly, it is not known which anti-VEGF agent is more appropriate in the treatment of ANAION. We elected to inject 2 mg of aflibercept intravitreally in the present case. To our best knowledge, our case is the first case of ANAION treated with aflibercept in the English-written literature.
